# Control of Autophagy in Cancer

**DOI:** 10.1155/2015/698740

**Published:** 2015-03-10

**Authors:** Arkadiusz Orzechowski, Saverio Bettuzzi, Patrycja Pawlikowska, Beata Pająk

**Affiliations:** ^1^Department of Physiological Sciences, Warsaw University of Life Sciences (SGGW), Nowoursynowska 159, 02-776 Warsaw, Poland; ^2^Electron Microscopy Platform, Mossakowski Medical Research Centre PAS, Pawińskiego 5, 02-106 Warsaw, Poland; ^3^Department of Biomedicine, Biotechnology and Translational Research, University of Parma, Via Volturno 39, 43100 Parma, Italy; ^4^Gustave Roussy Cancer Campus, 114 Rue Edouard Saillant, 94805 Villejuif, France

Autophagy (macroautophagy) is a self-degradative physiological mechanism observed in insufficiently nourished or injured cells and often activated to meet energy requirements or for cleaning up damaged organelles. Cancer cells, which are known to require more energy and nutrients than benign counterparts, may eventually take advantage of autophagy for survival. This special issue of this journal provides an updated view on the role of autophagy in the pathogenesis of cancer. The expert authors who have been invited to help with this task will describe the molecular mechanisms of autophagy with a special focus on tumor cells biology, dealing with those natural compounds which have potential anticancer activity because of being known to induce or inhibit autophagy.

The review entitled “Tumor Suppression and Promotion by Autophagy” addresses the issue of how autophagy affects tumorigenesis. It is apparent from several studies that tumor suppressor genes that negatively regulate mTOR, such as PTEN, AMPK, LKB1, and TSC1/2, stimulate autophagy. Conversely, oncogenes that activate mTOR, such as class I PI3K, Ras, Rheb, and AKT, inhibit autophagy. All together this suggests that autophagy is a tumor suppressor mechanism. Nevertheless, autophagy also functions as a cytoprotective mechanism under stress conditions, including hypoxia and nutrient starvation. These phenomena may promote tumor growth and induce resistance to chemotherapy in established tumors.

Another review entitled “The Importance of Autophagy Regulation in Breast Cancer Development and Treatment” summarizes the current knowledge on autophagy regulation in breast cancer, describing up-to-date anticancer strategies correlated with autophagy. During breast cancer development autophagy exerts different effects at cancer initiation and progression due to superimposition of signaling pathways of autophagy and carcinogenesis. Inhibition of autophagy may enhance the effectiveness of currently used anticancer drugs and other therapies (like radiotherapy). However, the promotion of autophagy can also induce death and, hence, elimination of cancer cells and reduction of tumor size. Thus, in the development of cancer, autophagy is regarded as a double-edged sword.

The review paper entitled “Calcium Homeostasis and ER Stress in Control of Autophagy in Cancer Cells” points to calcium ion homeostasis and starvation as the major factors influencing autophagy in tumors. Several Ca^2+^ channels like voltage-gated T- and L-type channels, IP3 receptors, or CRAC are involved in autophagy regulation as well as glucose transporters, mainly from GLUT family, which are often upregulated in cancer. Signals from both Ca^2+^ perturbations and glucose transport blockage might be integrated at UPR and ER stress activation. Thus modulation of autophagy might be a promising anticancer therapy. However, whether inhibition or activation of autophagy leads to tumor cell death or not appears to be a context-dependent matter.

The review entitled “Roles of Autophagy Induced by Natural Compounds in Prostate Cancer” focuses on prostate cancer (PCa), one of the most common cancers in aged men. Natural compounds showing low toxicity to benign tissue associated with specific anticancer effects at physiological levels* in vivo* are receiving increasing attention for prevention and/or treatment of PCa. Current evidence shows that some natural compounds may exert their action by modulating autophagy in PCa cells. Since mTOR activity can be directly or indirectly modulated by a number of upstream signaling pathways, it is mandatory to uncover the mechanisms through which these natural compounds inhibit the Akt/mTOR pathway and regulate the cell fate.

The review “Elaborating the Role of Natural Products-Induced Autophagy in Cancer Treatment: Achievements and Artifacts in the State of the Art” illustrates how the tumor suppressive action of natural products-induced autophagy may lead to cell senescence and apoptosis-independent cell death, also inducing complement apoptotic cell death by robust target-specific mechanism. Technicalities at detecting autophagy may affect the quality of the data; therefore it is suggested that rational criteria should be set up for monitoring natural products-induced autophagy in cancer cells. The action of autophagy-inducing natural products should be highlighted in future studies because it could become clinically relevant.

The paper entitled “Nucleofection of Rat Pheochromocytoma PC-12 Cells with Human Mutated Beta-Amyloid Precursor Protein Gene (*APP-sw*) Leads to Reduced Viability, Autophagy-Like Process, and Increased Expression and Secretion of Beta Amyloid” describes observations obtained from tumor pheochromocytoma PC-12 cells. These cells (immune to apoptosis) became sensitive to cell death following human* GFP* vector +* APP-sw* gene expression. Reduced cell viability was accompanied by higher expression of A*β* 1–16 and elevated secretion of A*β* 1–40. At the ultrastructural level autophagy-like process was demonstrated to occur in* APP-sw*-nucleofected cells with numerous autophagosomes and multivesicular bodies but without autolysosomes. Summing up, human* APP-sw* gene is harmful to PC-12 cells and cells are additionally driven to incomplete autophagy-like process.

In the following paper entitled “Combined Epidermal Growth Factor Receptor and Beclin1 Autophagic Protein Expression Analysis Identifies Different Clinical Presentations, Responses to Chemo- and Radiotherapy, and Prognosis in Glioblastoma” the authors investigated the expression of EGFR and Beclin1 in 117 glioblastoma undergoing postoperative chemo- or radiotherapy. Clinical cases are classified according to the level of expression of EGFR and Beclin1 and compared with clinical data. It is suggested that low expression of EGFR associated with high expression of Beclin1 could be a useful biomarker for the identification of a patient subgroup with relatively favorable clinical presentations and prognosis. This information supports the rationale for possible combined EGFR/Beclin1-targeted therapies.

Finally, the article “Gene Network Exploration of Crosstalk between Apoptosis and Autophagy in Chronic Myelogenous Leukemia” renders a graphical illustration of the most relevant gene networks for the exploration of functional links and potential coordinated regulations of gene expression related to apoptosis and autophagy in CML. In the CML-specific network, the link between E2F3 and AKT3 demonstrated a possible cell response to oncogenic stress which is active during the proliferation of hematopoietic cells. It is important to note that E2F3 and AKT3 were both the predicted targets of miR-15, whose deletion was proved to be associated with cancer promotion. The central role of E2F2 was further confirmed by the normal-specific transcription factor regulatory signature network. In the normal-specific miRNA regulatory signature network, the apoptotic balance was strengthened by the coregulation of BAK1 and BCL2 by miRNAs. As a normal-specific composite regulatory signature, the E2F2-BAK1-PIK3R5 motif may constitute the core mechanism controlling cell cycle progression, apoptosis, and autophagy. This hypothesis is worth further investigations in the future.

Overall, the current issue of this journal highlights the contribution of autophagy in tumorigenesis and describes which natural compounds are more promising in the future for chemoprevention and anticancer therapy on the basis of their ability to modulate autophagy.

*Arkadiusz Orzechowski* *Saverio Bettuzzi* *Patrycja Pawlikowska* *Beata Pająk*

